# A Case of a Female Patient Presenting With Idiopathic Granulomatous Mastitis With Superimposed Enterococcus Avium Infection

**DOI:** 10.7759/cureus.29997

**Published:** 2022-10-06

**Authors:** Mahdi Albogami, Abdulrahman Alsaedy, Rashed A Matrood, Yousef Alotaibi, Bushra Al Ahmadi

**Affiliations:** 1 College of Medicine, King Saud Bin Abdulaziz University for Health Sciences, Riyadh, SAU; 2 Department of Infectious Diseases, King Abdulaziz Medical City Riyadh, Riyadh, SAU; 3 Department of Pathology and Laboratory Medicine, King Abdulaziz Medical City, Riyadh, SAU

**Keywords:** chronic inflammation, non-necrotizing granulomatous, breast lump, enterococcus avium, idiopathic granulomatous mastitis (igm)

## Abstract

Idiopathic granulomatous mastitis (IGM) is defined as an uncommon, benign, chronic inflammatory disease of unknown etiology that affects the breast and can mimic breast cancer. It usually manifests as a solid, ill-defined breast lump in postpartum women of reproductive age. Furthermore, because it lacks a particular radiographic finding, core biopsy and histology are the only ways to make a conclusive diagnosis. There is no agreement on the best way to treat IGM. Ideally, a multidisciplinary approach should be used to weigh the benefits and drawbacks of each treatment option, with options such as observation, antibiotics, surgery, and medication therapy being examined (steroids and immunosuppressants). In this report, we review a case of a patient who had IGM with superimposed *Enterococcus avium* infection. To our knowledge, such a report is considered unprecedented in the Middle East. A literature review on IGM will be presented, as well as the clinical presentation, association with bacterial infection, treatment, and pathological and radiographic findings.

## Introduction

*Enterococci *are common gram-positive pathogens found in intestinal flora. Some enterococcal species cause serious illnesses. Clinical symptoms include endocarditis, intra-abdominal infections, and urinary tract infections, whereas soft tissue and bone infections are uncommon [1]. Because infections caused by *Enterococcus avium* account for approximately 1% of all human infections, only a few case reports of *E. avium* infection have been published [2]. Idiopathic granulomatous mastitis (IGM) is an uncommon, benign, chronic inflammatory disease of unknown etiology that affects the breast and can mimic malignancy [3]. IGM is uncommon in pregnant women, but it is common in other women of reproductive age, particularly from 6 months to 6 years postpartum. Increased levels of prolactin in the blood and an immunological response to local lobular secretions may play a role in pathogenesis because of their relationship with breastfeeding [4]. It usually manifests as a solid, ill-defined breast lump [3]. The lump usually appears unilaterally in any quadrant of the breast and is accompanied by discomfort and lymphadenopathy [5,6]. Nipple retraction, skin changes, and nipple discharge can occur, but they are uncommon. The mass may be complicated by abscess formation [6]. In this case report, we describe a case of IGM associated with *E. avium *infection in a Saudi woman. To our knowledge, this is the first case report of IGM associated with *E. avium* infection in the Middle East. The rareness of *E. avium* infection, as well as its unusual appearance and high degree of radiological overlap with breast cancer, increases its novelty and clinical significance.

## Case presentation

A previously healthy 46-year-old Saudi woman visited the emergency room complaining of pain and a mass in her right breast. She first noticed the mass almost one month ago, and it had been expanding rapidly and becoming more tender. A mammogram performed in another facility showed findings suspicious of cancer (BI-RADS 5). She had nipple retraction, but no fever, redness, or discharge. She was a nonsmoker and did not drink alcohol. She was breastfeeding and taking oral contraceptives. She had a history of mastitis secondary to milk stasis, which had been treated with antibiotics. Her parity was 7+2, and she had undergone a cesarean section three years ago with no complications. There was no family history of breast, colon, ovarian, or prostate cancer.

**Figure 1:**
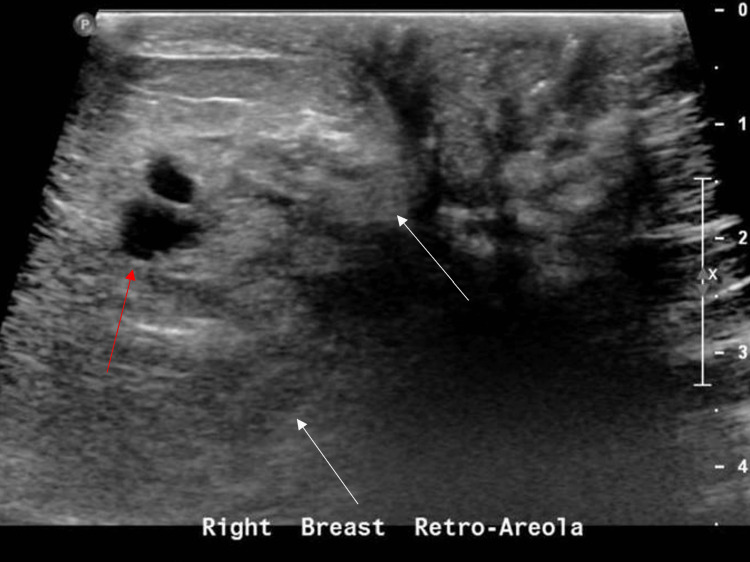
Ultrasound of the right breast retro-areola showing moderate swelling as well as heterogenous breast changes (white arrows). Focal areas of questionable nodules are seen in the inner aspect (red arrows).

At the breast surgery clinic, both her vital signs and laboratory workup were within the normal range. Physical examination revealed slight peau d’orange changes, skin thickening, a retracted nipple, and a 5 × 4 cm retro-areolar mass with no palpable axillary lymph node. A mammogram was requested, and it showed an incomplete evaluation of the right breast (BI-RADS 0), and a normal left breast (BI-RADS 1). Breast ultrasound revealed changes in the right breast that showed a predominantly inflammatory pattern; however, an associated malignancy could not be excluded (BI-RADS 4B), and thus right axillary lymph node and right breast biopsies were suggested (Figure 1).

**Figure 2:**
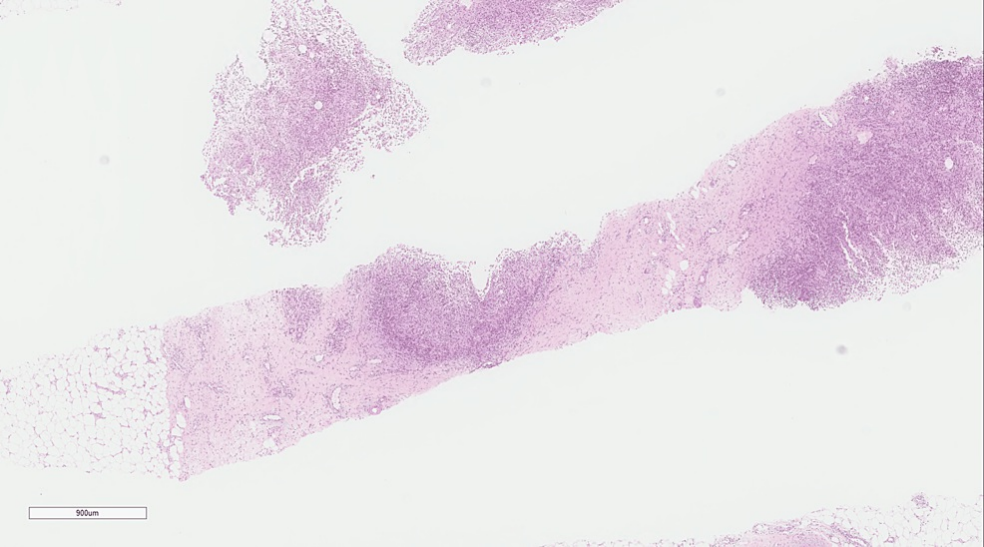
Low-power magnification of a core needle biopsy specimen of breast tissue showing multiple granulomas (hematoxylin and eosin stain, original magnification x 100).

**Figure 3:**
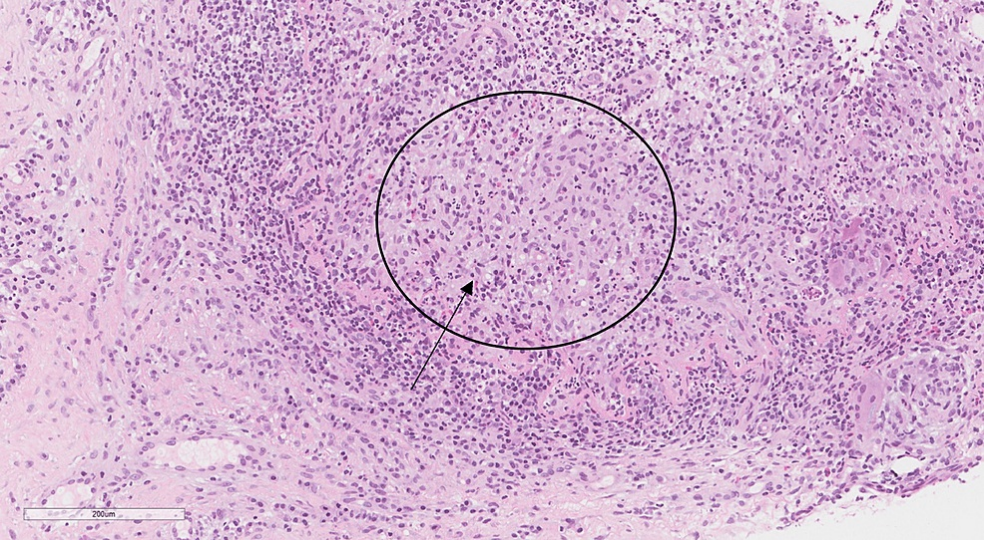
Higher-power magnification of a breast tissue biopsy specimen showing non-necrotizing granulomatous inflammation centered on lobules (black arrow), with lymphocytes, plasma cells, epithelioid histiocytes, scattered multinucleated giant cells, and neutrophils (hematoxylin and eosin stain, original magnification x 400).

A right axillary lymph node biopsy revealed reactive lymphoid tissue, and a core needle biopsy of the right breast showed non-necrotizing granulomatous inflammation, centered on the lobules, with lymphocytes, plasma cells, epithelioid histiocytes, scattered multinucleated giant cells, and neutrophils (Figures 2, 3). A polymerase chain reaction (PCR) test for tuberculosis (TB) was negative; therefore, all the findings suggested lobular IGM, which is a diagnosis of exclusion.

 

The patient was started on clindamycin (450 mg) and ciprofloxacin (500 mg) for seven days. After one week, she appeared healthy with no fever, redness, or abscess, but complained of pain at the site of the biopsy, and thus the antibiotics were continued for an additional two weeks. The patient was still experiencing pain and had signs of a small abscess. Culture of a fine-needle aspirate of the abscess showed *E. avium* organism, which was susceptible to ampicillin and gentamicin. The antibiotics were changed from clindamycin and ciprofloxacin to amoxicillin/clavulanic acid 625 mg orally for 14 days. The symptoms resolved one week later. Breast ultrasound revealed no evidence of an abscess. The antibiotics were discontinued, and the patient was reassured and discharged. She was advised to undergo an annual MRI indefinitely.

## Discussion

Clinical features

IGM typically presents in women aged 30-45 years as a mass with oblique margins in the upper outer quadrant of the breast. The lesion typically appears in one breast. It can occur in any quadrant, but primarily in the upper outer quadrant [5]. The lump is usually soft and may be accompanied by erythema, peau d’orange changes, and nipple inversion, which can be difficult to distinguish from breast cancer [7]. Abscess, ulceration, and fistula or sinus formation are common in severe or chronic cases [8]. In our case, the patient presented with pain, mass, and nipple retraction with slight peau d’orange in the right breast without skin inflammation or nipple discharge. The mass was 5 × 4 cm in size, located in the inner upper quadrant, and was associated with slight thickening of the overlying skin. Two weeks after the initial presentation, the clinical signs were similar, with the addition of a small abscess.

Imaging

IGM has no pathognomonic features on mammography, and mild lesions are frequently concealed by the relatively dense breast tissue [9]. Mammography typically reveals a mass with an uneven density and irregular boundaries. In addition, localized skin thickening, nipple inversion, and axillary lymphadenopathy contribute to the heterogeneity of the masses [10]. The most prevalent abnormalities in mammography are architectural distortion and irregular masses or nodules [9]. In our patient, mammography showed incomplete evaluation of the right breast (BI-RADS 0), and a normal left breast (BI-RADS 1).

In IGM, breast ultrasound can help with diagnosis. In more than 80% of cases, it reveals a heterogeneously hypoechoic mass lesion [9]. It can also show irregular masses, structural distortion, parenchymal edema, effusion, skin thickening, and enlargement of axillary lymph nodes, all of which are nonspecific findings [11]. In our patient, ultrasound showed moderate swelling of the retro-areolar region of the right breast, heterogeneous breast changes, and a prominent stromal pattern. Focal areas of possible nodules were seen in the inner aspect, with stromal changes. The changes in the right breast on ultrasound showed a predominantly inflammatory pattern; however, associated malignancy could not be excluded (BI-RADS 4B).

Histology

Histological analysis of tissue obtained from an open or a core needle biopsy is used to reach a conclusive diagnosis of IGM [12]. Fine needle aspiration biopsy is the least intrusive procedure, although it can produce false negative results, necessitating confirmation by open or core needle biopsy [13,14]. Histologically, IGM is characterized by lobulocentric noncaseating granulomas comprising epithelioid histiocytes mixed with Langhan’s multinucleate giant cells. Lymphocytes, plasma cells, and polymorphonuclear leukocytes are seen if chronic inflammation is present. In our case, core needle biopsy revealed multiple granulomas. The lesions showed non-necrotizing granulomatous inflammation centered on lobules, with lymphocytes, plasma cells, epithelioid histiocytes, scattered multinucleated giant cells, and neutrophils. In IGM, the lack of microorganisms on staining for bacteria and fungi is a key characteristic that rules out infective granulomas. The absence of caseous necrosis, with granulomas affecting lobules rather than ducts, differentiates IGM from TB. However, TB can be difficult to distinguish from IGM if the culture is negative for mycobacteria [10]. In our case, a PCR test of a biopsy specimen was negative for TB.

Microorganism

IGM is sometimes associated with bacterial infections, with gram-positive bacteria being the most common bacteria identified [15]. Some studies have found a relationship between IGM and *Corynebacterium* infection, suggesting that *Corynebacterium* could play a role in IGM [15,16]. One study found *Corynebacterium* in the breast tissue of 35 (55%) of 64 patients with IGM, and was more common in patients with fever or an abscess [15]. Another study evaluated the relationship between bacteriology and histologic diagnosis and found that 9 of 12 women with *Corynebacterium* infection had IGM pathologically [15]. A third study found that the effect of *Corynebacterium* infection in IGM may be linked to the infiltration of polymorphonuclear leukocytes in response to infection [16]. Moreover, *Corynebacterium* can be isolated from normal breast tissue [16]. In our case, two weeks after the initial presentation, the patient developed a small abscess, and culture of a pus sample revealed *E. avium* organism. To our knowledge, this is the first case to show an association between IGM and *E. avium* infection.

Treatment

There is no consensus on the best method for treating IGM. Ideally, a multidisciplinary approach should be used to weigh the benefits and risks of each treatment option. The treatment options include observation, antibiotics, surgery, and medication (steroids and immunosuppressants). In as many as half of the cases, IGM may become a chronic illness. Surgical therapy should be undertaken with caution, as the potential risks may outweigh the potential benefits [17,10]. In recent years, there has been a shift from total excision as the first-line treatment, with surgical intervention limited to biopsy, abscess drainage, and difficult fistula excision. Surgical excision, with or without supplementary steroid administration, is an acceptable and successful treatment option, when appropriate [18].

Antibiotics have a limited therapeutic effect on IGM. Only 3% of individuals who receive antibiotics show improvement in their condition [19]. However, our patient was administered clindamycin 450 mg and ciprofloxacin 500 mg for three weeks and then underwent fine-needle aspiration of a small abscess. The aspirate showed *E. avium* organism, which was susceptible to ampicillin and gentamicin; therefore, the antibiotic was switched to amoxicillin/clavulanic acid 625 mg orally. A week later, the patient’s symptoms improved, and thus the antibiotics were discontinued.

## Conclusions

In conclusion, the etiology of IGM remains unclear. Hence, a very high index of suspicion is essential to reach the diagnosis, which must be confirmed by core needle biopsy since IGM does not have specific radiological features. A link between bacterial infection and IGM has been suggested. To our knowledge, this is the first report of IGM associated with *E. avium* infection.
